# Editorial: Insights in cardiac rhythmology 2023

**DOI:** 10.3389/fcvm.2025.1542308

**Published:** 2025-01-17

**Authors:** Carola Griffith Brookles, Stefano Ruffini, Matteo Anselmino

**Affiliations:** ^1^ Division of Cardiology, Cardiovascular and Thoracic Department, “Città della Salute e della Scienza” Hospital, Turin, Italy; ^2^ Department of Medical Sciences, University of Turin, Turin, Italy

**Keywords:** atrial fibrillation, catheter ablation, autonomic nervous system, arrhythmic risk stratification, cardiac resynchronisation therapy

**Editorial on the Research Topic**
Insights in cardiac rhythmology 2023

It has been a dense year worldwide, as geopolitical instability, economic uncertainty and effects of climate change are building up pressure on most societies. Similarly, the scientific World is faced by many upcoming challenges: an ageing population with increasing comorbidities, as much as the constant growth of new technologies whose economic costs are likely to burden on stretched sanitary budgets (1–3.) It is essential to respond to these challenges by strengthening the efforts to guarantee necessary treatments remain accessible and affordable to all patients (4).

**Figure 1:**
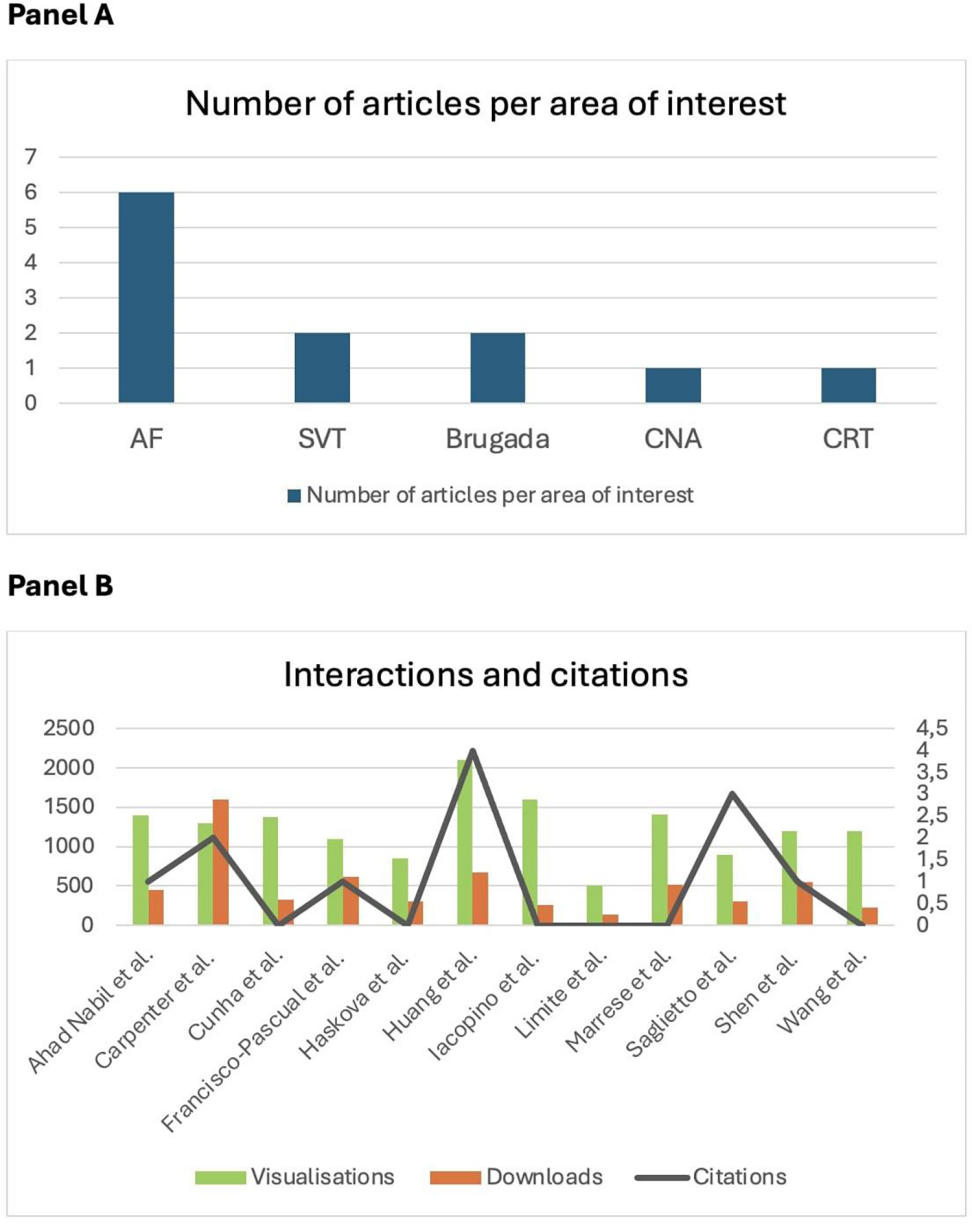
**(A)** Number of articles per area of interest published in Frontiers in Cardiovascular Medicine: Insights in Cardiac Rhythmology 2023. Atrial fibrillation (AF) gets the lion's share of the clinical research, in accordance with it's greater prevalence and clinical relevance. SVT, supraventricular tachycardia; CAN, cardioneuroablation; CRT, cardiac resynchronisation therapy. **(B)** Number of interactions as visualisations and downloads (left) and citations (right) of papers published in Frontiers in Cardiovascular Medicine: Insights in cardiac rhythmology 2023, by the end of November 2024.

In Cardiac Rhythmology, this year once again underscored the great interest in atrial fibrillation (AF) (Figure 1), with an expanding role for catheter ablation (CA) to reduce symptoms, recurrence and progression. As a result, the number of procedures is expected to rise steadily in the next years, also as a first line approach (5, 6).

Currently, CA demonstrates a high success rate and low incidence of complications (7), but trans-septal puncture (TSP) remains one of the most challenging steps of this procedure, also exposing the patients to an associated radiation risk (8). A retrospective analysis (Silva Cunha et al.) demonstrated the possibility to reduce fluoroscopy time by a single compared to a double TSP approach, with similar rates of efficacy and complications at follow-up.

CA outcomes are less satisfactory in patients with persistent AF (perAF) and extensive atrial remodeling. In the pre-pacific study (Limite et al.), patients with perAF who had a positive response to electrical cardioversion (ECV) were found to have less extended abnormal left atrial substrate at voltage mapping. A more aggressive approach beyond pulmonary vein isolation (PVI) in patients with ECV failure, displaying more advanced atrial remodeling, could therefore increase the success rate of CA (9). Convergent or hybrid AF ablation, that combines endocardial and mini-invasive epicardial approaches, has the potential to become a strategy to achieve rhythm control in these patients (10). When performed in experienced centers, the procedure was safe, with few and minor complications, as well as favorable in terms of rhythm control rates (Carpenter et al.)

Another recent innovation in the field of CA is represented by pulsed field ablation (PFA) (11). Interestingly, this technique has shown potential applications outside the field of AF. In a case report of a young man with repetitive episodes of orthodromic atrio-ventricular re-entrant tachycardia (AVRT) refractory to previous endocardial ablation attempts, PFA was successfully adopted for accessory pathway ablation, avoiding epicardial access and the related higher risk of complications.

An increasing awareness of the role played by the autonomic nervous system (ANS) in both the development and persistence of AF is emerging (Huang et al.). Autonomic dysregulation altering the electrophysiological properties of the atrium, through direct effects on ion channel function and disrupted calcium handling, can lead to atrial tachyarrhythmias. Interestingly, an imbalance between sympathetic and parasympathetic activities from acute injury to central autonomic regions, such as the insular cortex, resulted in an increased risk of cardiovascular and arrhythmic complications. Despite the complexity of the brain-heart connection, these findings suggest that therapies targeting the central nervous system and upstream pathways may represent a breakthrough in the treatment of AF.

Also dietary patterns, such as the Mediterranean and DASH (Dietary Approaches to Stop Hypertension) diets, play a role in modulating AF risk and progression. Incorporating nutrients like omega-3 fatty acids, magnesium and antioxidants might reduce incidence and progression of the arrhythmia, as part of a more holistic approach (Ahad Nabil et al.)

One of the most challenging aspects regarding AF is unfolding the relationship with cardioembolic ischemic events (12). Therefore, the possibility to identify a radiologic fingerprint to predict a cardio-embolic genesis of stroke would have a major impact on clinical practice, with a potential influence on secondary prevention strategies. Considering CA as an “*in vivo*” model of cardioembolic lesions, Saglietto et al. described core radiologic features supportive of a cardioembolic origin: cortical location in the territory of the middle cerebral artery, small dimensions (< 10 mm) and ubiquitarian distribution among lobes.

Shifting the attention from AF to bradyarrhythmias, cardioneuroablation (CNA) has emerged as a promising therapeutic option for young patients with recurrent cardioinhibitory syncopes that are unresponsive to conservative measures and in whom implantation of a permanent pacemaker is undesirable (13). Implementation of patients’ selection (through head-up tilt test, atropine test and Holter-monitoring derived indexes such as heart rate variability and deceleration capacity), as well as the diffusion of techniques to accurately identify the ganglionic plexi location during the procedure have prompted the diffusion of the technique. However, the lack of long-term follow-up and randomized controlled trials leave uncertainties regarding the extension of ablation (whether directed towards only right or both atria) and the definition of procedural endpoints (Marrese et al.)

Focusing the attention on inherited disorders, stratification and management of arrhythmic risk still represents a major challenge, as unexpected triggers might lead to fatal events. In a recently published case-report, lacosamide (an anti-epileptic drug which acts as an enhancer of slow-inactivated state of neuronal voltage-gated sodium channels) triggered the development of ventricular fibrillation in a young Asian female with seizures. After the detection of a type 1 Brugada pattern during ajmaline test, genetic testing revealed the presence of a heterozygous mutation in the SCN5A gene.

Considering the role of depolarization abnormalities in arrhythmogenesis in Brugada syndrome (14, 15), combination of novel ECG markers (dST-Tiso interval—the interval between the onset of the coved ST elevation and its termination on the isoelectric line) and ECG imaging (ECGi) through CardioInsight (a non-invasive 3D mapping system) have been tested to stratify arrhythmic risk in a 48-year old patient with drug-induced type 1 Brugada pattern and an anamnesis of syncope (Iacopino et al.), unveiling the presence of a conduction block along the anterior wall of the right ventricular outflow tract during ajmaline infusion. A subsequent electrophysiological study resulted positive for induction of VF, leading to the implantation of a single chamber defibrillator. Despite requiring further validation, ECGi may be useful for non-invasive stratification of arrhythmic risk in such patients.

An interventional approach with CA has become the first-line treatment in patients with congenital heart diseases with supraventricular and ventricular arrhythmias, as antiarrhythmic drugs can be problematic in this category (Francisco-Pascual et al.). In such patients meticulous procedure planning is essential, taking into consideration the patient's specific anatomy to identify a suitable vascular access. Intraprocedural imaging techniques, as well as the fusion of electroanatomic mapping (EAM) with previous imaging testing can provide guidance for a successful intervention.

Eventually, there is a persisting effort to better predict response to well-established treatment, such as resynchronization therapy (CRT). In a prospective study (Wang et al.), lateral wall regional constructive work (CW) and septal wasted work (WW) were identified as independent determinants of reverse modelling, with a positive impact on clinical outcomes and a better performance than global parameters.

This year's research confirms that cardiac rhythmology is indeed a fascinating and ever-evolving field! Despite these exciting developments, many questions still lay unanswered as new evidence continuously emerge. We can only look forward to the next year being a turning one for the many unresolved issues, in and outside of the scientific world.
